# AI-based biplane X-ray image-guided method for distal radius fracture reduction

**DOI:** 10.3389/fbioe.2025.1502669

**Published:** 2025-02-19

**Authors:** Qing Zha, Sizhou Shen, Ziyang Ma, Manqiu Yu, Hongzheng Bi, Hongbo Yang

**Affiliations:** ^1^ School of Biomedical Engineering (Suzhou), Division of Life Sciences and Medicine, University of Science and Technology of China, Hefei, China; ^2^ Suzhou Institute of Biomedical Engineering and Technology, Chinese Academy of Science, Suzhou, China; ^3^ Institute for Translational Medicine, Shanghai University, Shanghai, China; ^4^ Orthopedic, Shandong Wendeng Orthopedic Hospital, Weihai, China

**Keywords:** distal radius fracture reduction, artificial intelligence, x-ray image-guided, parameters computation, UNET

## Abstract

**Background:**

In the course of manual reduction of distal radius fractures, many doctors rely on tactile perception to assess the displacement of the fracture. However, a more accurate determination of the severity of the fracture and the success of the reduction requires measurement software to annotate the fracture images, which is difficult to achieve real-timely in actual procedure of reduction. Which may lead to misdiagnosis when experienced doctors rely on their intuition. Therefore, it is necessary to develop an AI-based method for calculating fracture parameters to provide real-time display, particularly in fracture reduction machines.

**Methods:**

An AI-based method for automatically calculating of radiographic parameters in distal radius fractures (DRF) was developed. Initially, anteroposterior (AP) and lateral (LAT) X-ray images of patients with distal radius fractures were collected from three hospitals and preprocessed. Subsequently, several neural network structures, UNet, DeeplabV3+, PSPNet, and TransUNet, are compared in terms of utility and accuracy, and finally, the models obtained from the UNet image segmentation algorithm are used for semantic segmentation of the radius and ulna. Following this, the contours of the radius and ulna were extracted using OpenCV, key points were detected, and the principal axes were calculated. Finally, the computed parameters including radial angle (RA), radial length (RL), ulnar variance (UV), and palmar tilt (PT) were calculated and displayed on the image.

**Results:**

The advantages and disadvantages of several models were considered, and finally the UNet neural network model was used as the core algorithm of the image segmentation model in this study. The segmentation accuracy for the radius and ulna in the AP and LAT X-ray images reached 91.31% and 88.63%, respectively. The average errors between the automated calculations of parameters RA, RL, UV, and PT and the manually annotated results by physicians were −1.36°, −1.7 mm, 0.66 mm, and −1.06°, respectively. The system has been initially deployed on the same computer that operates the radial fracture fracture repositioning robot.

**Conclusion:**

The automated parameter calculation method developed in this study accurately computes diagnostic parameters for assessing distal radius fractures and can be utilized in the image-guided reduction process of fracture rehabilitation robots. This method has the potential to evolve into an intelligent diagnostic tool for physicians, thereby enhancing the accuracy of distal radius fracture diagnosis.

## 1 Introduction

Hospitals worldwide receive a substantial number of patients with fractures every year, among which distal radius fractures are one of the most prevalent, accounting for 18% of all fracture incidents (Court-Brown and Caesar, 2006). The rapid and accurate assessment of fracture status and the formulation of appropriate treatment plans is crucial. Currently, most hospitals primarily rely on the subjective diagnosis of specialized physicians and manual reduction rehabilitation for the diagnosis and treatment of DRFs. Given the complexity of the pathology and the shortage of specialized medical professionals (Hoyler et al., 2014; Zhang et al., 2020), there has been a growing body of research in recent years focused on automated fracture detection (Zhao et al., 2020), many deep learning-based models have shown significant progress in improving diagnostic accuracy. For instance, researchers such as Gan et al. (2019) employed the Inception-v4 model to analyze 2,340 wrist X-rays, combined with the Faster R-CNN object detection algorithm to locate the distal radius region in the images, achieving an AUC of 96%. Chung et al. (2018) applied a basic CNN to analyze 1,891 shoulder X-rays, achieving an impressive 99.6% accuracy in fracture detection. Raisuddin et al. (2021) introduced the Grad-Cam module (similar to attention mechanisms) and achieved 95% accuracy in detecting fractures in the AP view and 98% in the LAT view using a dataset of 3,873 X-rays. On the other hand, Jabbar et al. (2022) augmented 193 original X-rays to 1,554 images and used an RN-21CNN network for wrist fracture detection, achieving a 97% accuracy, outperforming four other transfer learning models, including Inceptionv3, Vgg16, ResNet-50, and Vgg19. Ahmed et al. (2024) used a one-stage YOLO model for wrist fracture detection, with YOLOv8 achieving an average detection accuracy of 95%, outperforming the commonly used two-stage Fast R-CNN detection algorithm, highlighting the potential of one-stage models in pediatric wrist imaging analysis. Thian et al. (2019), based on the Faster R-CNN model, trained on 7,356 X-ray images and validated on 524 test images, achieved fracture detection accuracy of 91.2% for AP and 96.3% for LAT views.

These studies highlight the impressive performance of convolutional neural networks (CNNs) in fracture detection (Esteva et al., 2019; Gu et al., 2018). Olczak et al. (2017) is the first to apply deep learning in an orthopedic context, utilizing various deep learning networks, including CNNs, for fracture identification. Kim and MacKinnon (2018) adapted a pre-trained deep convolutional neural network model, originally designed for non-medical images, to a smaller dataset of X-ray images for automated fracture detection. Yoon et al. (2021) achieved detection of occult fractures that are invisible to human observers through deep convolutional neural networks (DCNNs). Additionally, Suzuki et al. (2022) applied CNN-based image classification for the diagnosis of distal radius fractures.

These findings demonstrate the powerful potential of CNNs for automated fracture detection and provide strong support for clinical applications. However, most studies focus solely on the detection of fractures, i.e., determining whether a fracture is present, without delving into the analysis of fracture severity. Therefore, while CNNs have made significant progress in fracture detection, further high-quality data and research are needed to support more comprehensive analysis of fracture complexity and the development of fracture reduction guidance technologies. Traditional treatment for distal radius fractures begins with the evaluation of radiological images to assess the fracture, followed by classification. For stable fractures, physicians typically rely on their clinical experience to compute key fracture parameters before performing manual reduction after local anesthesia, ultimately stabilizing the fracture site. Fracture reduction is a critical step in fracture management, as it involves repositioning the fracture ends to facilitate healing. During this process, image-guided technology is increasingly applied to improve procedural accuracy (Kraus et al., 2012). Leung et al. (2010) demonstrate a navigation function for fracture surgeries utilizing real-time fluoroscopic images and optical tracking systems. Furthermore, preoperative calculations of certain skeletal parameters (Kordon et al., 2020) can aid physicians in formulating more precise surgical plans, which is an important adjunct in orthopedic procedures, as anatomical parameters of the distal radius are significant in treatment selection (Bilgin et al., 2023). Currently, physicians typically rely on manual outlining of fracture parameters from X-ray images (Watanabe, 2019), combined with their experience to determine reduction techniques. This process is not only cumbersome but also highly dependent on the physician’s expertise. Therefore, automating the calculation of fracture parameters from imaging data and selecting the most suitable reduction technique has become an urgent issue. Suna et al. (2023) designed a pipeline capable of automatically calculating six fracture parameters from X-ray images; however, not all parameters are essential for fracture reduction in practical applications.

To address these issues, we propose a method for the automatic calculation of distal radius fracture parameters based on the UNet architecture. Due to its unique network structure, UNet can achieve detailed feature extraction from relatively small annotated datasets (Azad et al., 2024), and it has been widely applied in medical image segmentation, such as cardiac segmentation in magnetic resonance imaging (MRI) (Yu et al., 2017) and organ segmentation in computed tomography (CT) scans (Yu et al., 2018). The proposed pipeline consists of two main steps: first, the separation of the radius and ulna in the AP and LAT X-ray images using UNet, followed by the calculation of key fracture parameters based on the segmented images. These parameters provide crucial data support for fracture reduction robots. By integrating robotic-assisted reduction, the goal is to advance the technology used in fracture treatment, enabling a more effective and precise reduction process.

## 2 Materials and methods

### 2.1 Dataset construction

**FIGURE 1:**
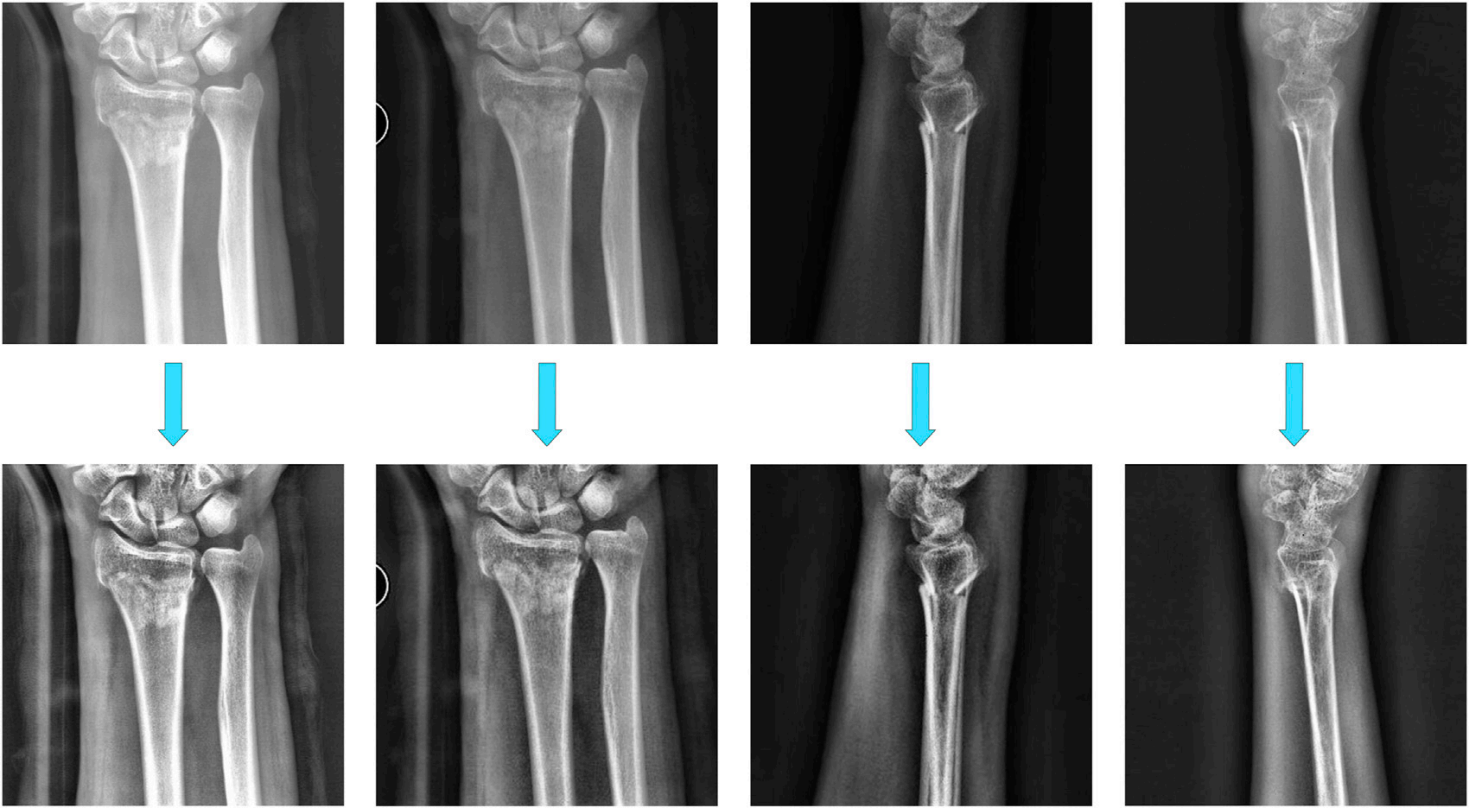
Histogram equalization of image.

In this study, we evaluated our method using a dataset of 441 X-ray image sets (each set includes one AP and one LAT image) from Suzhou Hospital of Traditional Chinese Medicine and Jiangsu Province Hospital of Chinese Medicine. The dataset comprises 371 fracture cases and 70 normal instances, all of which have been approved by the local ethics committee. After preliminary screening, some images are excluded due to issues such as poor imaging quality or incorrect wrist positioning, which will affect diagnostic accuracy. While the hospital-provided images facilitate fracture assessment for clinicians and specialists, consistent image processing is required for image recognition systems, including standardizing image size, wrist proportion, and rotation angle to ensure accurate automated identification and analysis. Before constructing the dataset, preprocessing image and augmentation is absolutely necessary, as depicted in the figure. Four images are shown that have been pre-processed and histograms are equalized in Figure 1. Histogram equalization is a straightforward and effective image enhancement technique, usually used to improve image quality and contrast by adjusting histogram shapes, which could help to reduce grayscale level differences between images, thereby simplifying the annotation and segmentation process. Subsequent testing demonstrated that the dataset processed with histogram equalization significantly improved model training success rates. The preprocessed images were used to create the dataset, which is annotated using the conventional tool Labelme (version 3.16.7). The annotations are saved as JSON files and then converted into mask images in the VOCdevkit format, facilitating subsequent model training.

### 2.2 Image segmentation

In the field of medical image segmentation, UNet has proven to be an extremely efficient model. Skeletal image segmentation provides material for subsequent parameter computation, and the specific segmentation model is as follows:

(1) A model for image segmentation of the distal radius, proximal radius, and entire ulna in orthopantomograms of the wrist joints (referred to as AP bone segmentation model).
(2) A model for image segmentation of the distal and proximal radius in lateral radiographs of the wrist joints (referred to as the LAT bone segmentation model).

**FIGURE 2:**
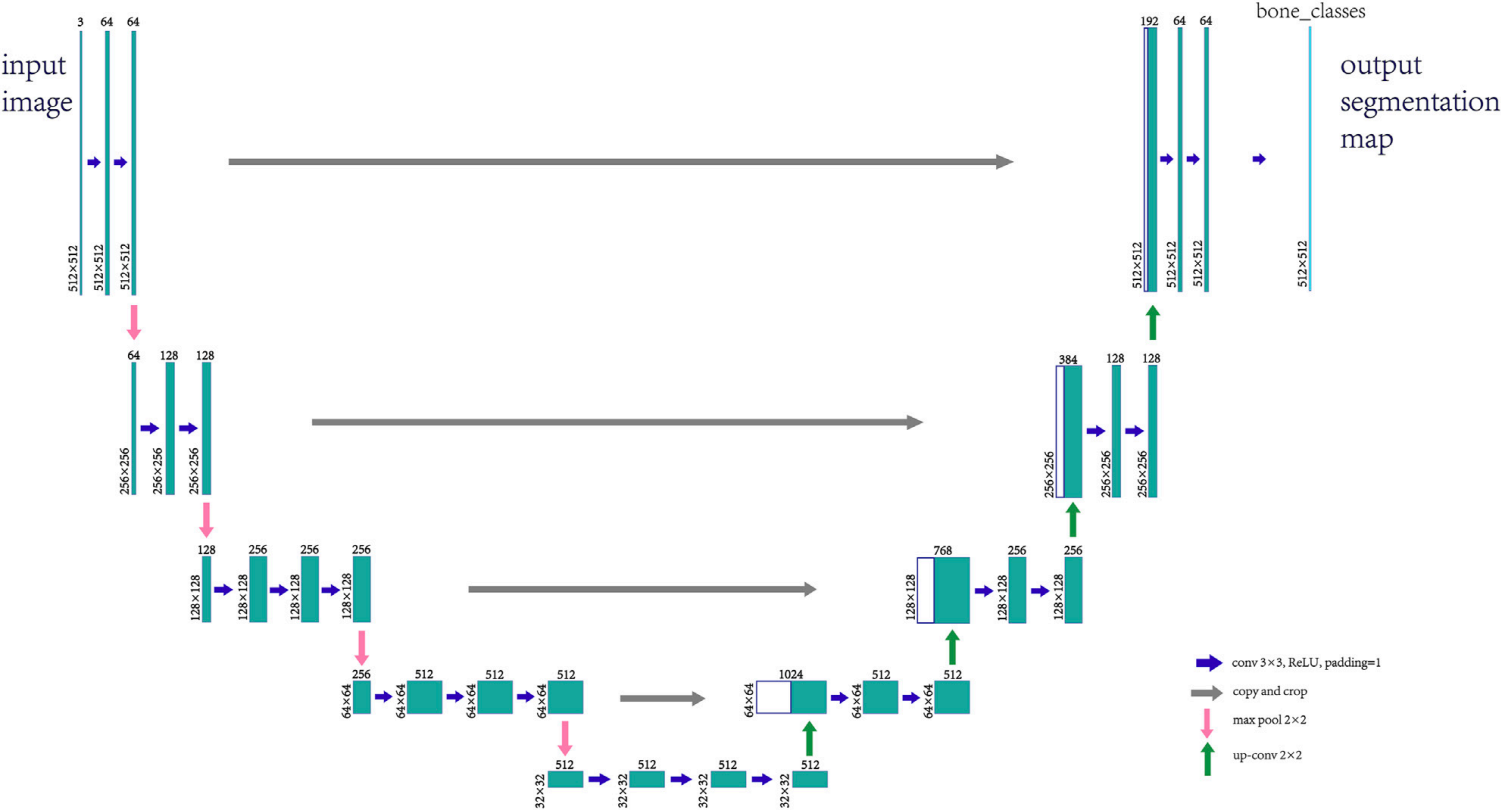
Feature extraction network.

The data preprocessing steps used for the dataset used in this step are as described above, and the final image input to the deep learning network should be a single channel image of (512, 512, 1) and the radial segmentation annotation is generated using Labelme. The specific composition of the UNet neural network used in this article is as follows:

(1) Backbone Feature Extraction Network: The part of the backbone feature extraction network is VGG16, which consists of convolution and maximum pooling, the convolution process uses (3*3) convolution kernel and sets the padding to 1, which is designed to ensure that the convolution process maintains the dimensionality of the image and reduces the loss of the edge information, and the specific backbone extraction network is shown in Figure 2. Five preliminary effective feature layers can be obtained by this backbone feature extraction network. The feature layer generated after the last convolution pooling operation in the backbone feature extraction network is (32, 32, 512).
(2) Enhanced Feature Extraction Network: The feature map obtained from the last convolutional pooling layer is doubly up-sampled. The doubly up-sampled feature map is then fused with the initial valid feature map obtained from the backbone feature extraction network, with both feature maps being stacked.

The self-constructed dataset will be inputted into the above feature extraction network for training; due to the limited size of the dataset of self-constructed skeletal X-ray images, if the training is started from 0, it may trigger the randomness of the parameter weights to be too large, which in turn negatively affects the model’s stability and convergence, therefore, we will use the same network as the feature extraction network, and use the publicly available dataset and the other self-constructed medicine class grayscale mapping dataset as the training set Therefore, the same network will be used as the feature extraction network, and the public dataset and other self-built pharmaceutical gray map datasets will be used as the training set to generate the pre-training weights, which will be used for migration learning in this study. The loss function of the model in this paper chooses the loss function system composed of Cross-Entropy Loss combined with Dice coefficients or Focal_Loss combined with Dice coefficients.

The above deep learning models were trained on different datasets for AP bone segmentation model and LAT bone segmentation model respectively. When training the LAT bone segmentation model, a loss function combining Cross-Entropy Loss and Dice coefficients is used; the pre-processed X-ray images are already normalized, and there is no positive or negative sample imbalance in each part, and for the focal loss function, the Cross-Entropy function does not require additional hyper-parameter adjustment, which reduces the model training. The complexity of model training is reduced.

Cross-Entropy Loss:

$$CE\left(p,y\right)=-\sum_{i=1}^{n}y_{i}\log\;\left(p_{i}\right)$$

where $p_{i}$ denotes the probability that the prediction is for class i and $y_{i}$ denotes whether class i is a true label.

While training the distal radius, proximal radius and ulna segmentation models, a fusion loss function composed of a focal loss function (Focal_Loss) combined with Dice coefficients is used. The dataset labels used to train this model have a large disparity in the number of pixels in each target category and a low sample balance, and the moderating factor added to the focal loss function solves the sample imbalance problem.

Focal_Loss:

$$FL\left(p_{t}\right)=-\alpha_{t}{\left(1-p_{t}\right)}^{y}\log\;\left(p_{t}\right)$$

where $p_{t}$ is the predictive probability of the model for the sample (for positive samples, $p_{t}=p$ ; for negative samples, $p_{t}=1-p$ ) and $\alpha_{t}$ is the category weight.

Dice_Coefficient:

$$Dice\_Coefficient=\frac{2\left|X\cap Y\right|+\varepsilon }{\left|X\right|+\left|Y\right|+\varepsilon }$$

Dice_Coefficient=1−Dice_Loss

$$Dice\_Loss=1-\frac{2\left|X\cap Y\right|+\varepsilon }{\left|X\right|+\left|Y\right|+\varepsilon }$$

where X denotes the pixel label of the true segmented image, Y denotes the pixel category of the model’s predicted segmented image, $\left|X\cap Y\right|$ is approximated as the dot product between the pixels of the predicted image and the pixels of the really labeled image, and $\left|X\right|$ and $\left|Y\right|$ are the summation of the pixels in their respective corresponding images, respectively.

$$Dice\_Loss=1-\frac{\left(1+\delta^{2}\right)TP+\varepsilon }{\left(1+\delta^{2}\right)TP+\delta^{2}FN+FP+\varepsilon }$$

$$\left|X\right|=TP+FN$$

$$\left|Y\right|=TP+FP$$

where TP (True Positives) means that the prediction is positive and actually positive; FP (False Positives) means that the prediction is positive but actually negative; FN (False Negatives) means that the prediction is negative but actually positive; TN (True Negatives) means that the prediction is negative and is actually negative; δ is a parameter used to adjust the trade-off between precision and recall; ε denotes a smoothing term that avoids the denominator being zero.

**FIGURE 3:**
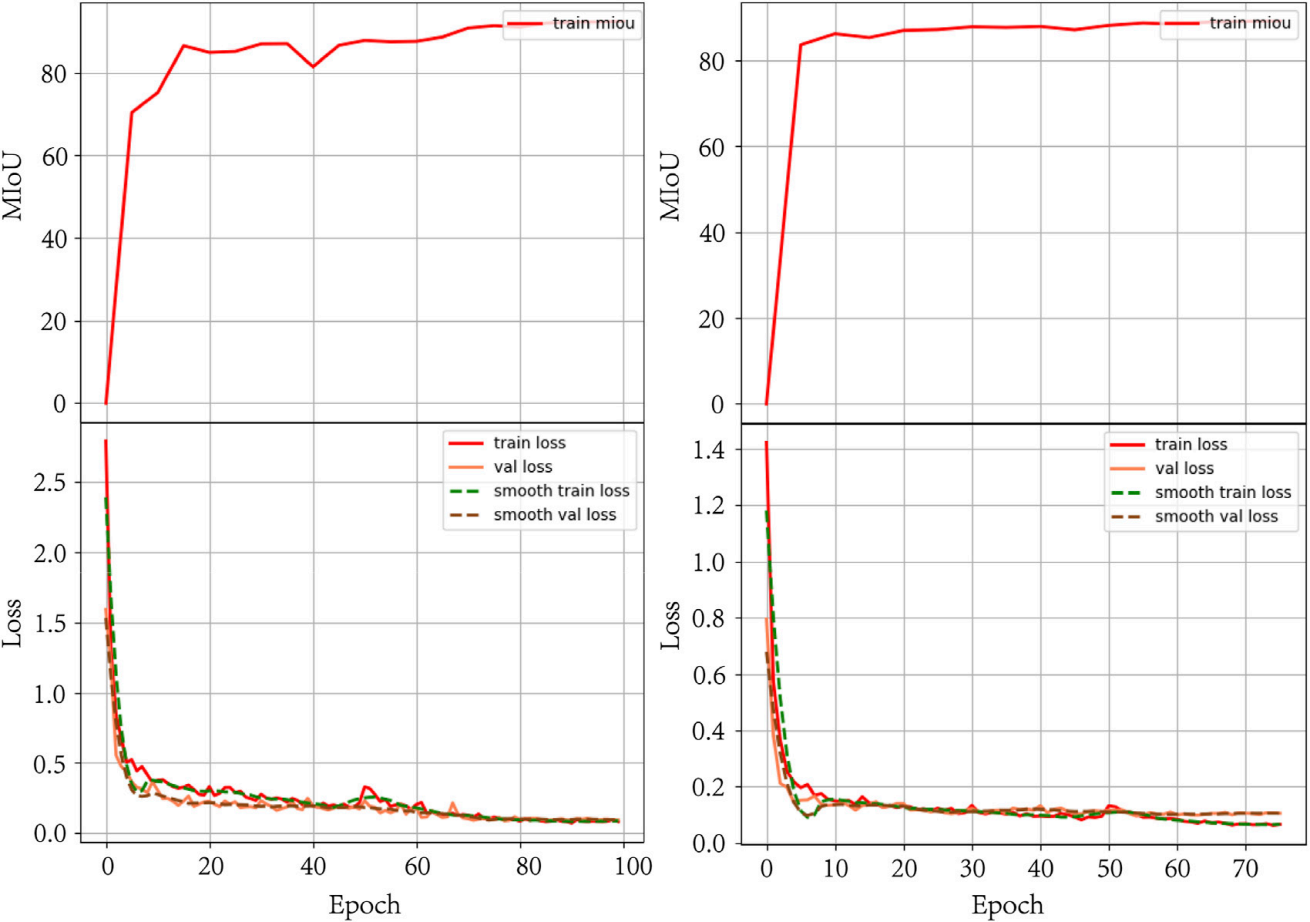
Line graphs of loss function values and changes in MIoU values.

**FIGURE 4:**
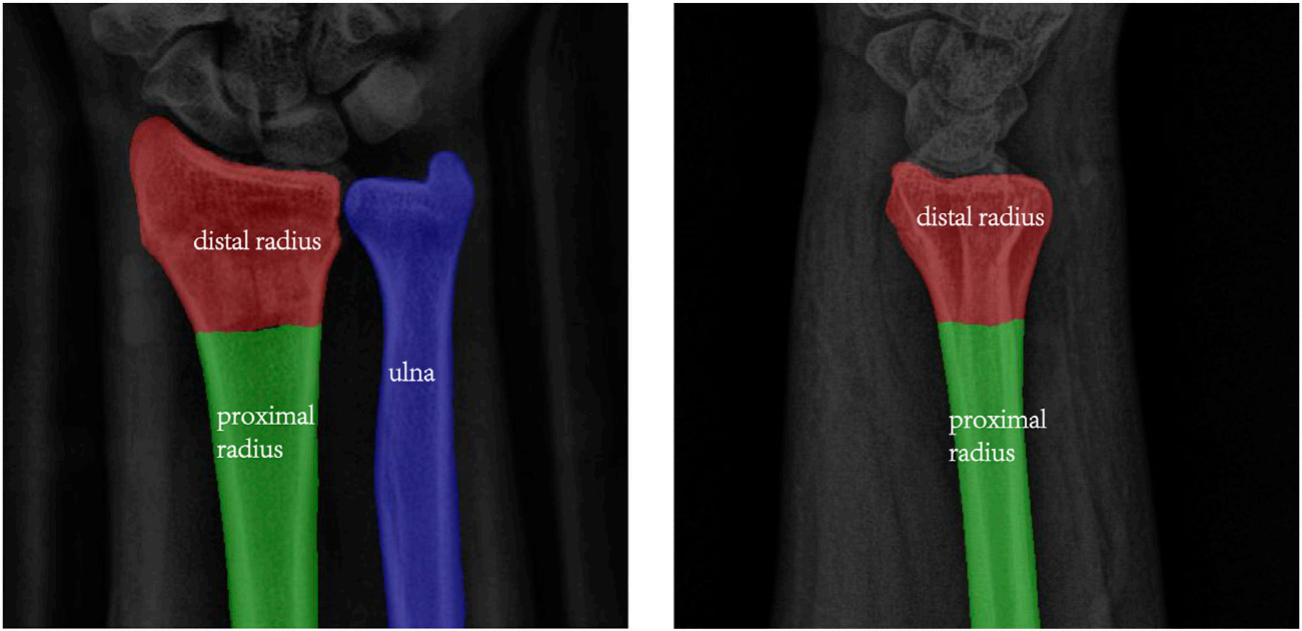
Segmentation results of AP image (left) and LAT image (right).

The changes in the loss function as well as the MIoU values during the training iterations of the above two models are shown in Figure 3. The figure shows that the loss function decreases with the number of iterations, and the MIoU value becomes larger with the number of iterations, which proves that the two models for this dataset should have converged, and after the number of iterations reaches 60 rounds, the curves of the loss function and the MIoU value tend to flatten, and there is a risk of overfitting if the training continues. In the process of two training iterations, the optimal one of each evaluation parameter that appeared during the model iteration was selected as the final model and predicted, and the segmentation was successfully completed as shown in Figure 4.

Although the UNet neural network is currently considered by many to be very suitable for medical image segmentation, in order to make our study more convincing, we used the PSPNet architecture model, which is also used for image segmentation, the DeeplabV3+ architecture model, as well as the TransUNet architecture neural network, which is also suitable for the field of medical image segmentation, to conduct comparative experiments.

The PSPNet network for the control experiments uses Mobilenetv2 as the backbone feature extraction network, and the backbone feature extraction network in this paper uses four downsamplings. The enhanced feature extraction structure uses the conventional PSP module, which divides the acquired feature layer into regions of different sizes, each of which is individually average pooled. The loss function of the PSPNet model chooses the loss function system composed of Cross- Entropy Loss combined with Dice coefficients or Focal_Loss combined with Dice coefficients. To serve as an experimental control group, the dataset used to train the model is the same as the one used to train the model for the UNet neural network and the training process is the same as the one used for the UNet neural network.

DeeplabV3+ architectural model as a control in this paper uses Mobilenetv2 as the backbone feature extraction network, to strengthen the feature extraction network focuses on Encoder species, the initial effective feature layer compressed four times using parallel Atrous Convolution, respectively, with different rates of Atrous Convolution for feature extraction, and then merge and convolution; in Decoder kind of the effective feature layer compressed twice using convolution to adjust the number of channels, and then stacked with the effective feature layer upsampling results after hollow convolution, and then two depth separable convolution blocks to obtain the final effective feature layer, the loss function is similar to the previous.

The TransUNet architecture neural network in this study serves as a control group, and its structure does not change, consisting of CNN, Transformer, and Decoder of UNet. The datasets used for training are also all the same as before.

### 2.3 Contour extraction

The calculation of key parameters for distal radius fractures involves using AP and LAT segmented images as input and producing corresponding fracture parameters as output. The pipeline consists of the following four steps: (1) Extract contour lines from the segmented images; (2) Determine the axis lines of the corresponding components; (3) Locate anatomical landmarks of the radial fracture; (4) Calculate radiographic parameters based on the identified key points and the axis lines of the radius and ulna, and subsequently assess the severity of the fracture.

In the contour line extraction step, we first process the segmented AP and LAT images. The detailed procedure is as follows:

(1) Mask Creation and Image Binarization: Create a mask based on different color information to separate the different parts of the radius and ulna. Perform flood filling from the coordinate origin outward to fill each part of the image. Then, apply bitwise operations to invert the image and convert it to a binary format.
(2) Image Denoising: Due to inherent errors in the image segmentation module, apply a 3 × 3 Gaussian kernel for denoising.

$$G\left(x,y\right)=\frac{1}{2\pi \sigma^{2}}\exp\;\left(-\frac{x^{2}+y^{2}}{2\sigma^{2}}\right)$$

where σ represents the standard deviation of the Gaussian kernel. This step smooths the boundaries of the segmented regions and enhances the accuracy of contour extraction.

(3) Edge Detection and Contour Extraction: Use the Sobel edge detection algorithm to extract edges from the binary image, which helps reduce computation and eliminate irrelevant information.

$$G_{x}=\left[\begin{array}{ccc} -1 & 0 & 1\\ -2 & 0 & 2\\ -1 & 0 & 1 \end{array}\right]$$

$$G_{y}=\left[\begin{array}{ccc} -1 & -2 & -1\\ 0 & 0 & 0\\ 1 & 2 & 1 \end{array}\right]$$

The gradients in the horizontal $G_{x}$ and vertical $G_{y}$ directions are calculated using convolution with the denoised image to approximate the intensity differences, thereby locating the edges and mitigating the impact of segmentation errors on contour extraction.

**FIGURE 5:**
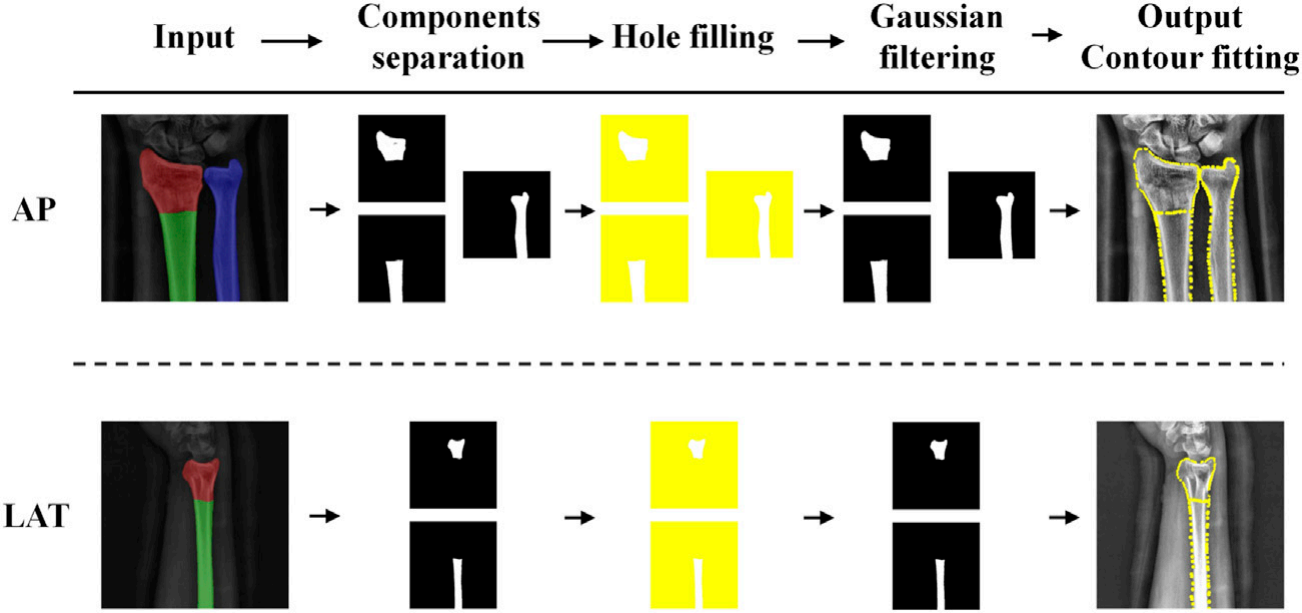
Pipeline of ulnar and radius contour line extraction.

(4) Contour Fitting and Optimization: Extract contours from the edge-detected image. Given that segmentation modules may have errors and even after hole filling, some mis-segmented regions may remain, sort all extracted contours and select the one with the largest area as the final contour. Use a triangulation-based contour fitting method to divide the contour into a series of triangles. Record the coordinates of each triangle’s vertices and their boundaries as follows:

$$V=\left\{\left(x_{1},y_{1}\right),\right.\left(x_{2},y_{2}\right),\ldots ,\left.\left(x_{n},y_{n}\right)\right\}$$

where $\left(x_{i},y_{i}\right)$ represent the coordinates of the *i*th vertex. Simplify the contour data by recording the direction and distance of movement from one vertex to the next, reducing the amount of data for subsequent calculations and improving image processing efficiency. Finally, a matrix composed of the coordinates of the contour points is obtained. Figure 5 shows the pipeline of extracting the contour lines of bones.

### 2.4 Calculation of central axis

To begin with, a set number of random points $\left(x_{i},y_{i}\right)$ (where i = 1, … ,n)are selected within the contour. For a given line equation y=mx+b , the distance $d_{i}$ from each point to this line is computed.

$$d_{i}\left.=\right|y_{i}-(mx_{i}\left.+b)\right|$$

where $d_{i}$ represents the Manhattan distance from the point $\left(x_{i},y_{i}\right)$ to the line. To smooth these distances, we use the following formula:

$$distance=2\left(\sqrt{1+\frac{d^{2}}{2}}-1\right)$$

where d is the Manhattan distance:

$$d=d_{i}$$

Substitute d into the formula, we obtain the adjusted distance from each point to the line:

$${adjusted\_distance}_{i}=2\left(\sqrt{1+\frac{{d_{i}}^{2}}{2}}-1\right)$$

This method of distance calculation preserves the robustness of the Manhattan distance while incorporating the smoothness of the Euclidean distance. This results in a better distribution of weights among the random points, and the fitted line more closely aligns with the central axis of the radius. The fitted line y=mx+b with the following losses:

$$Axis\_Loss=\sum_{i=1}^{n}distance\left(x_{i},y_{i},m,b\right)$$

where $\left(x_{i},y_{i}\right)$ are the coordinates of the random points within the radius contour.

To find the line parameters m and b that minimize the loss function, we use gradient descent optimization. We start with initial values $\left(m_{0},b_{0}\right)$ for the line parameters, typical 0. Then update m and b by calculating the gradient values:

$$m_{new}=m-\alpha \frac{\partial Axis\_Loss}{\partial m}$$

$$b_{new}=b-\alpha \frac{\partial Axis\_Loss}{\partial b}$$

Where α is the learning rate that determines the size of each update step.

By repeatedly calculating the loss function, computing gradients, and updating the parameters using gradient descent, we adjust the parameters until the loss function is sufficiently minimized. This yields the axis lines for the radius and ulna in the AP and LAT images, respectively. The axis line from the frontal image of the ulna is used as a reference axis for further calculation of fracture parameters.

### 2.5 Key point extraction

Distal radius fractures often cause significant damage to the structure of the ulna and radius. To accurately characterize the severity of these fractures, we need to define a set of parameters. Key parameters include ulnar variance, radial height, volar angulation, and ulnar variance. Based on the unique anatomy of the radius and the required parameters, we identify the following five key points:

(1) Styloid Tip (ST): In the AP image, the highest point of the radial styloid is commonly used to assess the relative height, angle, and rotation of the radius. After segmenting the radius using deep learning methods, we create a color mask to obtain an array of pixel coordinates for the radius’s contour. Considering the differences in image reading directions by the computer, we locate the ST by finding the pixel with the smallest vertical coordinate among these points, which is identified based on the vertical coordinate of the contour pixel points.
(2) Ulna Border of Radius (UBR): Located at the intersection of the distal radial ulnar joint surface and the ulnar joint surface in the anteroposterior image. The relative position of this point to the ST helps determine the radius’s relative height and deformation. We create a line through the ST, starting with a slope of 0. Using ST as the rotation center and rotating clockwise by 0.01° increments, we continue until the line intersects the radius contour at the first point. If this intersection is significantly distant from ST, it is considered the UBR, if not, we adjust the line until an appropriate UBR point is found and obtain its position coordinates.
(3) Ulnar Height (UH): Located on the flat joint surface of the distal ulna in the AP image. This point is used to evaluate the relative height of the ulna to the radius joint surface. First, determine the highest point on the ulna contour and use it as the rotation center. Rotate a line counterclockwise until it intersects the ulna contour at the first point, denoted as ul_UBR (the ulnar border of the radius on the ulna). Next, find the intersection of the ulna axis with the ulna contour, ensuring that all contour points between this intersection and ul_UBR lie on the flat joint surface of the distal ulna. The midpoint of these contour points is UH.
(4) Dorsal Joint (DJ): Located at the highest point of the dorsal joint surface of the distal radius in the lateral image. Using the lateral image segmented by deep learning, the highest point of the radius is identified as DJ.
(5) Volar Joint Border (VJB): Located at the highest point of the volar joint surface of the distal radius in the LAT image. After finding DJ, determine whether the fracture end has shifted volarly or dorsally. If volar, rotate a line through DJ counterclockwise with DJ as the rotation center until it intersects the radius contour at the first point. If dorsal, rotate the line clockwise until it intersects the radius contour at the first point. This intersection point is identified as the VJB.

### 2.6 Calculation of fracture radiographic parameters

**FIGURE 6:**
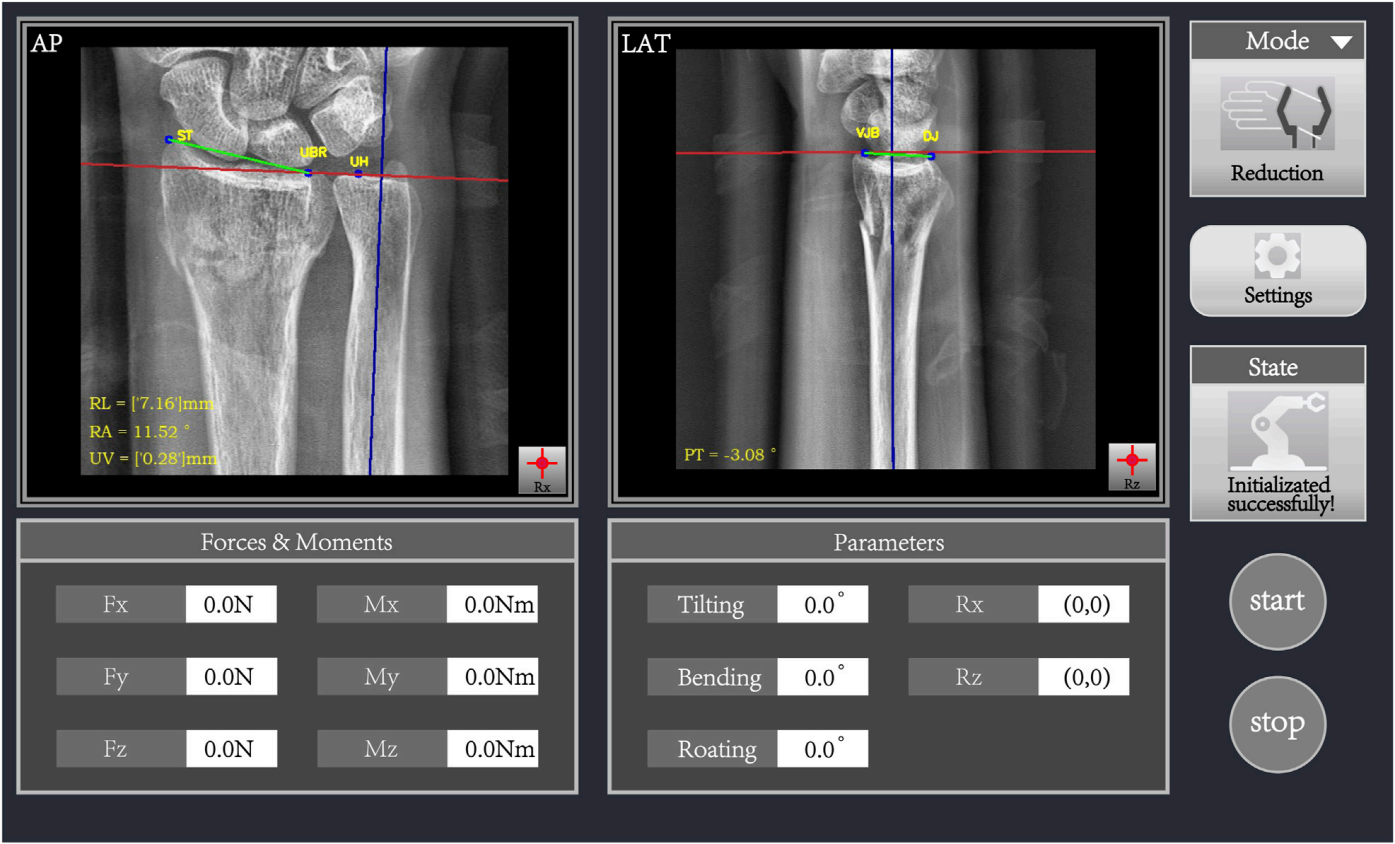
Auto-scribing results of AP image (left) and LAT image (right).

Parameters related to distal radius fractures are crucial for diagnosing the fracture, selecting treatment options, assessing prognosis, and guiding rehabilitation. Figure 6 shows the results of auto-scribing for different images.

#### 2.6.1 Radial Angle

This parameter describes the angle between the distal radius, the wrist bones, and the metacarpals. It is an important indicator for assessing the type of distal radius fracture (e.g., Colles or Barton), as different fracture types can lead to significant changes in RA. The calculation process for RA is as follows:

(1) parameter identification: First, identify two key points: the ST and the UBR. The ST is the highest point of the radial styloid, while the UBR is located at the intersection of the distal radial ulnar joint surface and the ulnar joint surface.
(2) Reference axis line: Establish a reference axis line perpendicular to the baseline axis through the UBR point.
(3) Angle calculation: Calculate the angle between the line connecting ST and UBR and the reference axis line. This angle is RA. The specific calculation formula is:
$$RA=\tan^{-1}\frac{y_{ST}-y_{UBR}}{x_{ST}-x_{UBR}}-Reference\_angle$$
  where $\left(x_{ST},y_{ST}\right)$ and $\left(x_{UBR},y_{UBR}\right)$ are the coordinates of ST and UBR, and Reference_angle represents the angle of the reference axis.

#### 2.6.2 Radial Length (RL)

This is an important parameter for describing the relative height of the radial styloid. The normal range is between 8 and 14 mm. Variations in radial height are significant for fracture classification and treatment planning. The calculation process for RL is as follows:

(1) Parallel lines establishment: Draw two parallel lines perpendicular to the baseline axis: one through ST and the other through UBR.
(2) Distance calculation: The distance between these two parallel lines represents RL. Since ST and UBR correspond to the highest point of the radial styloid and the ulnar border of the radius, respectively, this distance accurately reflects the relative height of the radius and helps determine the degree of radial shortening. The formula for this calculation is:
$$RL=distance\left({Line}_{ST},{Line}_{UBR}\right)$$
  where $distance\left({Line}_{ST},{Line}_{UBR}\right)$ represents the vertical distance between the parallel lines drawn through ST and UBR.

#### 2.6.3 Ulnar Variance

Malunion of distal radius fractures can lead to positive ulnar variance, increasing contact between the ulna and the lunate and triquetrum bones. This can result in wrist pain and adversely affect the recovery process. The calculation of UV is as follows:

(1) Establishment of parallel lines Draw two perpendicular lines to the baseline axis at the distal end of the ulnar joint surface and at the ulnar notch of the radius, passing through the UH and UBR points, respectively.
(2) Distance calculation: The distance between these two perpendicular lines represents UV. The formula for calculating UV is:
$$UV=distance\left({Line}_{UV},{Line}_{UBR}\right)$$
  where $distance\left({Line}_{UV},{Line}_{UBR}\right)$ represents the vertical distance between the parallel lines drawn through UV and UBR.

#### 2.6.4 Palmar Tilt (PT)

The angle between the line connecting the volar and dorsal extremes of the radius in the lateral image and the perpendicular to the baseline axis in the LAT image.

(1) Parameter identification: First, identify the two key points: the VJB and DJ.
(2) Reference axis establishment: Establish the reference axis as the perpendicular line to the long axis of the radius at its distal end in the LAT image.
(3) Angle calculation: Calculate the angle between the line connecting VJB and DJ and the reference axis. This angle is referred to as the volar angulation.

#### 2.6.5 Volar Joint Border (VJB)

Located at the highest point of the volar joint surface of the distal radius in the LAT image. After finding DJ, determine whether the fracture end has shifted volarly or dorsally. If volar, rotate a line through DJ counterclockwise with DJ as the rotation center until it intersects the radius contour at the first point. If dorsal, rotate the line clockwise until it intersects the radius contour at the first point. This intersection point is identified as the VJB.

## 3 Results

### 3.1 Segmentation accuracy of ulna and radius images

Skeletal segmentation modeling is the first step in this study, and this step has a huge impact on the accuracy of the subsequent steps such as parameter computation, and the previous chapters have described in detail the steps of dataset production, feature network selection, loss function selection, and model training in the training of the model in this study. In this study, the quality of the bone segmentation model is evaluated by the four parameters mIoU (mean Intersection over Union), mPA (mean Pixel Accuracy), mPrecision, and mRecall, which are calculated by running the trained model on the test set and calculated. Table 1 shows the values of each parameter used to assess the model quality calculated by calling the models after running on the test set, from the parameter values we can see that the AP bone segmentation is of higher quality, and each parameter used to assess the model quality of the AP bone segmentation model is better than that of the LAT bone segmentation model, and the AP bone segmentation model is more accurate than the LAT bone segmentation, which is supposed to be the result of the overlap of radius-ulnar in the LAT view and the approximation of various features of the ulna. It is supposed that the overlap of the radius-ulna in the LAT view and the approximation of the radius characteristics lead to the larger error of the LAT bone segmentation model.

**TABLE 1:** Evaluation of image segmentation models based on different neural network training (UNet, PSPUNet, DeeplabV3+, TransUNet).

Models	mIoU (%)	mPA (%)	mPrecision (%)	mRecall (%)
UNet (AP/LAT)	91.31/88.63	96.27/95.47	94.56/92.42	96.27/95.47
PSPNet (AP/LAT)	85.96/82.55	89.14/88.33	90.95/89.27	89.04/86.33
DeeplabV3+(AP/LAT)	86.99/83.38	91.94/89.17	92.11/90.86	92.23/89.18
TransUNet (AP/LAT)	94.84/88.31	95.63/94.05	96.28/93.22	95.83/93.78

From the longitudinal comparison of the model evaluation data in Table 1, it can be seen that the models trained based on UNet or TransUNet are significantly better than the other two, while the evaluation indexes of UNet and TransUNet are superior and the difference is very small. However, two problems were encountered in the implementation process, firstly, the neural network of TransUNet architecture requires a large amount of computation during the model training process, which is more demanding on the hardware equipment, and the training models in this study were all trained on a computer equipped with a 12th Gen Intel(R) Core(TM) i7-12700k processor and an NVDIA GeForce RTX3090 chip, and the length of training a single UNet-based network architecture and TransUNet-based network architecture is 55 min and 72 min, respectively, using the same number of iterations and datasets. The system studied in this paper is not only adapted to the corresponding surgical robots, but also may need to be adapted to different x-ray machines under many conditions, which means that in order to adapt to different x-ray machines, the system needs to be modified. This means that in order to adapt to different x-ray machines, the model needs to be trained specifically, and the huge amount of computation is not conducive to the practical application of the system and the surgical robot; and when the system is mounted on the surgical robot, it needs to display the progress of the surgery and the various parameters in real time, which requires the model to be lightweight, and the parameters of the model obtained through the TransUNet architecture are large. In the test set of this study, the average time for the model trained on TransUNet neural network to predict a single AP and LAT angle image is 1.12s and 0.85s, respectively, while the average time for the model trained on UNet neural network to predict a AP and LAT image is 0.45s and 0.36s, respectively, and the delay in the display of parameter computation caused by the larger delay in the segmentation of the image is not favorable to timely help doctors to obtain the condition information.

In summary, at this stage of the study, the UNet neural network is still the optimal solution.

### 3.2 Comparison of fracture parameter calculations

By comparing the fracture parameters calculated by physicians with those obtained using the automated landmarking system, statistical analysis indicates that the differences between the two sets of measurements are not statistically significant. This suggests that the proposed pipeline does not exhibit systematic errors in parameter computation, demonstrating its high reliability and potential for clinical adoption.

Specifically, we conducted a detailed statistical analysis of the differences in four major fracture parameters. For the RA parameter, the mean difference was −1.36° (SD = 0.267), indicating a slight negative bias in the angles calculated by the automated system compared to those computed by physicians. However, the small standard deviation (0.267°) and lack of statistical significance suggest that this bias is minimal and unlikely to affect clinical decision-making. For the RL parameter, the mean difference was −1.7 mm (SD = 0.489), which, while slightly negative, remained within the clinically acceptable range of variation, with a small standard deviation indicating minimal variability. The UV parameter showed a mean difference of 0.66 mm (SD = 0.120), reflecting a minimal and stable error, with high consistency across multiple test samples. For the PT parameter, the mean difference was −1.06° (SD = 0.148), also indicating a slight negative bias but with minimal fluctuation.

**FIGURE 7:**
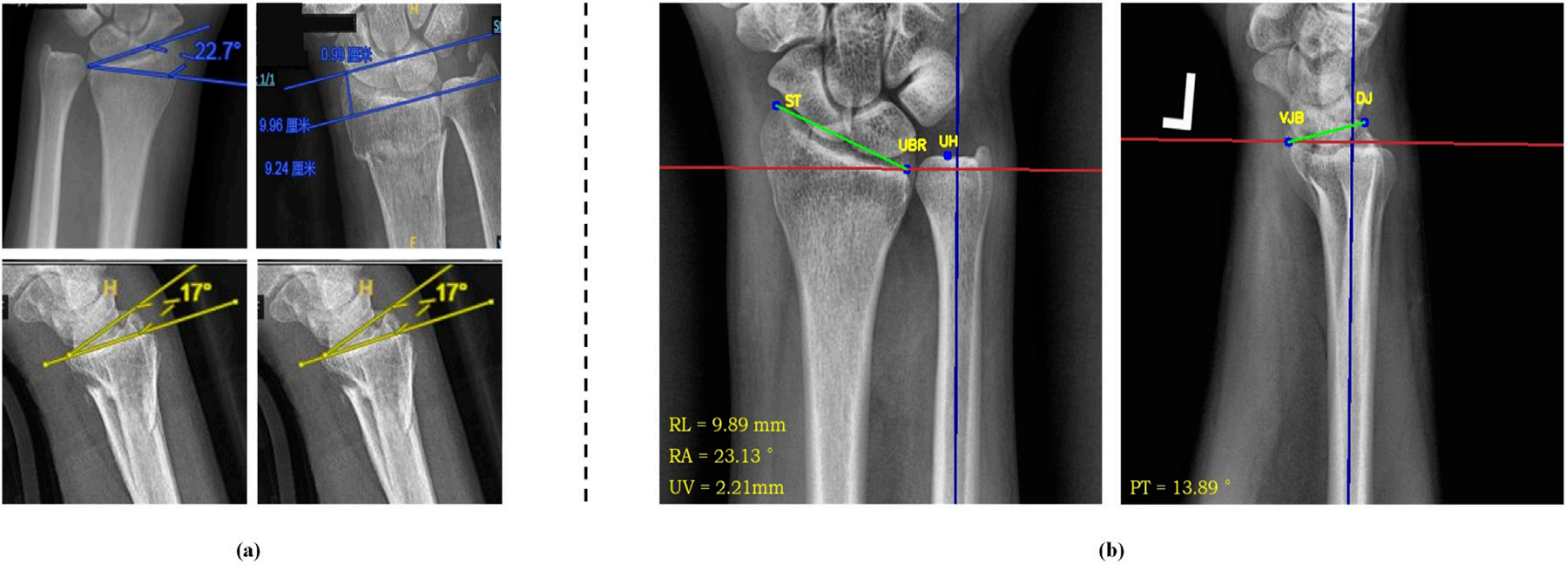
Comparison of manual annotation **(A)** and automatic computation **(B)** results.

To evaluate the robustness of the system, the analysis was repeated on an independent dataset, showing consistent results with a similar range of differences. Overall, the automated fracture parameter calculation system demonstrates high accuracy and consistency, with errors that are statistically insignificant when compared to manual measurements by physicians. The observed mean differences in RA, RL, and PT parameters fall within the clinically accepted thresholds for fracture assessment, suggesting that the automated system could be used in clinical practice without significant risk of misdiagnosis. The results indicate that our system offers a reliable alternative to manual annotation, with minimal impact on clinical outcomes. Figure 7 presents the differences between manually annotated calculations (a) and automatically computed parameters (b). It can be observed that the discrepancies in RA, RL, and UV are minimal, while the difference in PT is 3.11°. This discrepancy may arise from subjective factors during manual annotation, calibration errors, or limitations in the precision of the measuring instruments. However, the difference remains within an acceptable range, demonstrating the system’s reliability and consistency across different test cases.

### 3.3 Image-guided system field test

In order to evaluate the performance of this system when applied with the radius fracture reduction robot, we combined the image guidance system with the radius fracture reduction robot, firstly, we took pictures of the volunteers through the special x-ray machine on the robot, and then imported them into the image guidance system for the image segmentation and parameter calculation, and then finally, we specified the corresponding auxiliary treatment plan through each parameter, and manipulated the robotic arm to complete the treatment, the results and the software operation interface are shown in Figure 6. The calculation results and the software operation interface of the robotic arm are shown in Figure 6. Finally, the degree of goodness of the test results is positively correlated with the accuracy of the parameters; compared with the radius fracture repositioning robot without the image guidance system, it is more preferable to refer to the parameters calculated by the system and then carry out the assisted treatment, which greatly reduces the workload of medical personnel while improving the accuracy.

## 4 Discussion

The manual calculation of fracture parameters by physicians is highly dependent on the experience and energy of the physician, and is therefore susceptible to the influence of the physician’s personal experience and subjective factors, which may lead to inconsistencies in the calculation results, thus affecting the diagnostic accuracy of the fracture and the selection of the subsequent treatment plan. In contrast, automated fracture parameter calculation methods can significantly improve the ease and accuracy of calculation, providing physicians with more objective and consistent fracture assessment results. Therefore, in this paper, we employ deep learning, computer vision and other methods to realize the auxiliary recognition of distal radius fracture, the automation of parameter calculation, and to provide image guidance for the fracture reduction robot’s distal radius fracture reduction surgery, when we apply the model in our fracture reduction robot’s accompanying software, and we can carry out the status of the fracture reduction in the surgical process through the image guide real-time observation, avoiding the bad results caused by delayed fracture information in conventional reduction surgery or in robotic reduction surgery.

A feature extraction network based on the UNet architecture was used for the image segmentation of AP and LAT wrist X-ray images. This network utilizes the UNet deep learning architecture, which is combined with a VGG16 encoder for feature extraction. The parameter calculation and image guidance modules are based on the results of image segmentation, and employ computer vision techniques in conjunction with geometric methods for precise parameter extraction. High precision was achieved in both image segmentation and parameter calculation, as evidenced by the Intersection over Union (IoU) scores and parameter accuracy metrics. The IoU values for the models segmenting AP and LAT X-ray images were 91.31% and 88.63% on the test set. And in the above has been through the image segmentation accuracy, model training time and model prediction time multiple perspectives will UNet network and the other three neural network architecture comparison, DeeplabV3 + and PSPUNet neural network corresponding to the model is not only in the accuracy of the lower, in the training time has no advantage, respectively, need to be 60 + min and nearly 50 min of training, the accuracy of the higher The training of the model based on the TransUNet architecture neural network takes much longer. The difference between the prediction time of nearly 1s for the model based on the TransUNet architecture neural network and less than 0.5s for the model trained on the UNet neural network architecture is huge, which determines the validity of the information obtained by the physician in clinical use. In this study, in the process of combining image segmentation and parameter calculation, the whole system is compressed to less than 0.6s for a single single image calculation process.

The calculation of four fracture parameters in this study relies on the accurate fitting of a reference axis, making it crucial to enhance the accuracy of axis fitting to improve overall computational precision. We optimized the contour before fitting the axis and minimized the loss function through iterative computation and updating of the axis parameters to improve axis fitting accuracy. In AP images, the ulnar diaphysis was selected as the reference axis due to its relative stability and lower impact from distal radial fractures, providing a more consistent and reliable reference. In LAT images, we used deep learning methods to separate the fractured radial end from the radial diaphysis, with the latter being less affected by the fracture and thus offering a higher reference value. This automated calculation method, which involves automatically locating anatomical landmarks to compute fracture parameters, significantly reduces subjective interference while maintaining computational accuracy. Additionally, the automated approach provides precise data support for fracture reduction robots, which is critical for achieving accurate fracture realignment. The robots can utilize these fracture parameters to determine optimal reduction paths and force applications, improving the effectiveness and accuracy of the reduction process.

Analysis results indicate that the errors produced by the automated fracture parameter calculation system are not statistically significant compared to manual calculations by physicians, further validating the system’s reliability and accuracy. The four fracture parameters calculated by the automated method adequately meet the data requirements for fracture reduction robots, providing a reliable basis for selecting appropriate reduction paths and force levels.

Many deep learning applications and studies on fractures were mentioned above, Gan et al. (2019) used Faster R-CNN to localize the fracture region, Kunihiro’s team (Oka et al., 2021) accomplished fracture diagnosis by using CNN neural network with high accuracy, and Raisuddin et al. (2021) used Grad-Cam module to achieve fracture detection in dual The dataset required for most of these studies is very large, which means that the training time required is long and there is a big resistance in the process of specialization or popularization of the technique, and the purpose of these studies is to identify “fracture or not”, which, in practice, can help doctors to identify fracture injuries to a certain extent. However, it cannot assist doctors in real-time fracture restoration surgery in practice. The network structure used in this study is more stable and requires a smaller dataset, and the system studied in this paper focuses on practicality, which makes it less costly to specialize the system for use with other surgical robots in the future. In other studies, most image segmentation requires hundreds or even thousands of images, and the required data set cost is huge. In this study, in the image specialization process, only a few dozens of images are needed to complete, and with a small dataset, for example, with the hardware configuration used in this study, the training duration of the specialization model can be compressed to less than 30 min, and the new model can be adapted to the corresponding x-ray machine. Compared with previous studies, we divided the distal radius fracture reduction process into two subtasks: “ulnar radius segmentation” and “parameter calculation”. This division effectively improves the accuracy and efficiency of each step, reduces the calculation time, and improves the precision of fracture reduction. Moreover, by combining this system with the robot and semi-following software, the fracture reduction status can be monitored in real time, avoiding the adverse consequences of delayed acquisition of fracture information in traditional surgery or robot-assisted reduction.

However, the image segmentation component of this study has certain limitations. Although the training set underwent some preprocessing, its accuracy may decrease slightly when segmenting X-ray images from other hospitals due to the predominance of images from the same X-ray machine. Similarly, using different X-ray machines with integrated fracture reduction robots may also reveal insufficient model tolerance. Future improvements should focus on enhancing feature extraction algorithms to further increase model segmentation accuracy, or optimizing image preprocessing processes to ensure consistency in input images. Additionally, designing specific feature extraction algorithms for different types of X-ray machines could further enhance segmentation and parameter calculation accuracy.

Overall, automated fracture parameter calculation methods demonstrate superior performance in improving computational accuracy and reducing subjective influence. They not only streamline the fracture assessment process but also enhance the precision of fracture treatment, providing a solid data foundation for the advancement of fracture reduction techniques. Future research should explore the potential of this method in other orthopedic applications and continue to refine computational algorithms to enhance their clinical applicability and accuracy.

## 5 Conclusion

In this study, we developed an automated system for analyzing distal radius fractures, encompassing both fracture region identification and key fracture parameters calculation. First, we construct an image dataset comprising 371 sets of fracture X-ray images and 70 sets of healthy control images, obtained from the hospital information platform. Necessary preprocessing steps, including image adjustment and augmentation, are performed to enhance the accuracy and reliability of subsequent model training. We then employ the UNet model for bone image segmentation, validating the segmentation accuracy of AP and LAT bone models from different perspectives, and optimize model performance using appropriate loss functions.

Based on the precise bone segmentation results, we developed an automated method for calculating fracture parameters, including RA, RL, UV, and PT, which are crucial for fracture diagnosis, treatment planning, and prognosis assessment. These parameters also guide fracture reduction robots in planning reduction techniques. Compared to manual calculations by physicians, our automated system demonstrated high precision and consistency across all parameters. Although minor deviations were observed, they were not statistically significant, indicating the system’s reliability and effectiveness in practical applications.

**FIGURE 8:**
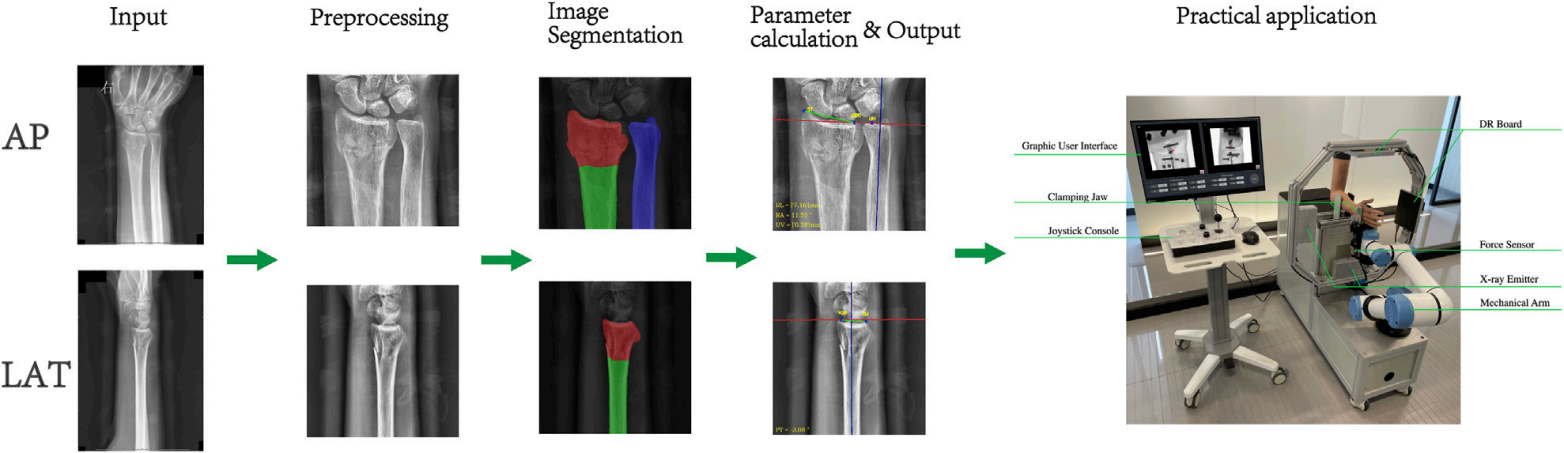
Image guidance system channel flow and system applications.

Overall, the automated system developed in this study not only improves the efficiency of fracture parameter calculations but also reduces the impact of subjective factors on diagnostic results, providing more objective and accurate data for fracture assessment and treatment. Furthermore, the system holds significant implications for the application of fracture reduction robots, offering precise reduction paths and force data, thereby enhancing reduction outcomes. Figure 8 has shown the flow path of the system channel and the final scenario of the initial application in combination with a radial repositioning robot. Future research can build on this foundation to further optimize model performance and calculation accuracy, aiming to make a more substantial contribution to the field of fracture diagnosis and treatment.

## Data Availability

The raw data supporting the conclusions of this article will be made available by the authors, without undue reservation.
